# Supplementary material for: Fifty Years of the National Rabies Control Program in Brazil under the One Health Perspective
Source: Pathogens. 2023 Nov 11;12(11):1342. doi: 10.3390/pathogens12111342 (PMC10674250; doi:10.3390/pathogens12111342)
Supplement: Supplementary file 1 [file pathogens-12-01342-s001.zip › pathogens-2690460-supplementary.pdf]

Table S1: Selected variables and sources of information used to create a database for the driver's analysis by municipality

Category	Variables	Period	Data sources
Outcome	Human rabies cases	2010–2022	Ministry of Health [1 2 3]
Demographic	Total population	2021	IBGE [4]
	% of rural population	2021	IBGE [4]
	Population density	2021	IBGE [4]
Environmental	Major habitat type [a]	-	FAO [5 6 7]
	Temperature [a]	1970–2000	WorldClim [8]
	Precipitation [a]	1970–2000	WorldClim [8]
	Forest loss [b]	2013-2017	Global Forest Change [9]
Socioeconomic	GDP per capita	2021	IBGE [10]
	Gini Index	2021	IBGE [10]
Covariables	Dog vaccination coverage	2012-2017	Ministry of Health [11]
	Basic health care coverage	2012-2017	Ministry of Health [12]

References:

[1] SINAN – Casos de raiva humana – Notificações registradas no sistema de informação de agravos de notificação Brasília, Brasil: Ministério da Saúde. 2023 [33]

[2] Ministério da Saude. Secretaria de Vigilância em Saude e Ambiente. Raiva. Situação epidemiológica. [34]
All human cases by the transmitting animal from 1986 to 2022 are available on tables and figures in the Ministry of Health webpage [34]. From 1973 to 1985, the source was Schneider (1990) based on the Ministry of Health. All canine cases of rabies from 2002 to 2020 are publicly available on the government website; before that, the source was Schneider (1990) and internal reports from the Ministry of Health [35].

[3] Schneider MC. Schneider MC. Estudo de avaliação sobre área de risco para a raiva no Brasil [Master dissertation]. Rio de Janeiros: Fundação Oswaldo Cruz. Escola Nacional de Saúde Pública; 1990. [35]

[4] IBGE - Fundação Instituto Brasileiro de Estatística. 2022. [30]

[5] FAO. FAO-GeoNetwork-geo-spatial - WWF Global Ecoregions Map [39].

[6] WWF Global - Major Habitat Types [40]

[7] United Nations Environment Programme (UNEP) [41]

[8] Fick SE, Hijmans RJ. Worldclim 2: New 1-km spatial resolution climate surfaces for global land areas. *International Journal of Climatology* 2017;37(12):4302-4315 [42]

[9] Hansen MC, Potapov PV, Moore R, Hancher M, Turubanova SA, Tyukavina A, et al. High-resolution global maps of 21st-century forest cover change. *Science* 2013;342:850–853 [43]

[10] IBGE - Fundação Instituto Brasileiro de Estatística. [44]

[11] Ministério da Saúde, Brasil. Informes sobre vacinação canina por município, 2012 a 2017 [internal document].

[12] Ministério da Saúde, Brasil. E-Gestor AB. Sistemas da Atenção Primaria a Saude. Histórico de Coberturas [32]

Legend:

[a] **Major habitat type, temperature, and precipitation**
Data preprocessing for these variables were the same methodology used in previous publication (Schneider MC et al. 2021 [38].According to FAO and WWF, eco-region units (867) are grouped into 14 major habitat types (MHT), which are defined as relatively large units of land or water containing a distinct assemblage of natural communities sharing a large majority of species, dynamics, climatic, and other environmental conditions.

[b] **Forest loss,**
Data preprocessing for Forest loss (or tree loss) was the same methodology used in previous publication (Min KD et al. 2019 [36], Schneider MC et al. 2021 [38].

Table S2: Definitions and procedures

Rabies prophylaxis	According to the Ministry of Health, the rabies vaccine (inactivated) is indicated for the prophylaxis of human rabies, being administered in individuals exposed to the rabies virus as a result of biting, licking of the mucous membrane, or scratching caused by transmitting animals, or as prophylaxis in people who, by their occupational activities, are permanently exposed to the risk of infection by the virus. In some situations, the indication of prophylaxis is complemented by the administration of serum [28].
Case of human rabies	According to the Ministry of Health guidelines [28], the following definitions are officially used in all health systems:<br><br>Suspected case: All patients with a clinical picture suggestive of encephalitis, with or without a history of exposure to rabies virus infection.<br><br>Confirmed case: This could be by laboratory criteria when a suspected case with compatible symptomatology has laboratory confirmation, for which IFD, or BP, or PCR, was positive for rabies. A case could also be confirmed by clinical-epidemiological criteria; when a patient with an acute neurological condition (encephalitis), who presents forms of hyperactivity, following of paralytic syndrome with progression to coma, without the possibility of laboratory diagnosis, but with a history of exposure to a likely source of infection.<br><br>Discarded cases: All suspected cases with negative IFD and BP or that, during the investigation, had their diagnosis laboratory confirmed by another etiology.<br><br>Notification: Every suspected human case of rabies is subject to compulsory and immediate notification at municipal, state and federal levels. The notification must be registered in the Notifiable Diseases Information System (SINAN), by completing and sending the Rabies Investigation Form.
Case of rabies in dogs and cats (rabies variants 1 and 2)	Suspected case: Any dog or cat that presents symptoms compatible with rabies, that dies from diseases with symptoms neurological disorders and for reasons to be clarified, with or without a history of aggression by another suspicious or rabid animal. Confirmed case: Any dog or cat suspected that, submitted to laboratory examination, reveals positivity for rabies, or every suspected dog or cat that has been clinically diagnosed as rabid by a veterinarian and evolved to death, even though no material was sent for laboratory diagnosis.<br><br>In case of detection of canine rabies, needs to immediately notify the case to the government at the municipal level (second subnational level) or

	state level. Together with the health authority, instructions will be given on how to proceed. Decisions and actions need to be made within 72 hours of notification; these include case investigation, situation analysis, and defined interventions. It is recommended that all cases of rabies in dogs and cats need to be sent samples to a reference laboratory for virus typification.
Dog vaccination coverage:	Dogs and cats are the targets of vaccination in Brazil; however, the major focus is dogs. The goal is to vaccinate 80% of the dog population. This study used the vaccination coverage of dog estimated population as a co-variable. This data was obtained by the Ministry of Health's internal report with the number of dogs vaccinated by municipalities and the estimated dog population. Different methods were used for the estimations of the dog population according to the state guidelines. The variable created to estimate vaccination coverage is the number of dogs vaccinated/number of dogs targeted)/*100. Eventually, the dog population estimation is not accurate; in the case of coverage of more than 100%, the data was adjusted to 100% by the authors.
Post-exposure prophylaxis	In Brazil, post-exposure prophylaxis (PEP) is free of charge in the Unified Health System. According to the Ministry of Health, rabies vaccine (inactivated) is indicated for the prophylaxis of human rabies, being administered in individuals exposed to the rabies virus as a result of biting, licking of the mucous membrane or scratching caused by transmitting animals, or as prophylaxis in people who, by their activities occupational, are permanently exposed to the risk of infection by the virus. In some situations, the indication of prophylaxis is complemented by the administration of serum [28].
Coverage of basic health care by the Unify Health System (SUS)	As part of SUS, the no-cost-to-public PEP could be administrated at vaccination rooms, hospitals, Emergency Care Units, and different types of Health Units, including Family Health Units. As a proxy of the access to PEP, an open access variable was used from an online Primary Care Coverage Report of the Ministry of Health (MS link report). This report includes data by municipality (second subnational level) of the estimated population coverage of Family Health Teams (in Portuguese Equipe de Saude da Familia -ESF). According to SUS strategy, the municipalities could have a Family Heath team, formed by a doctor, nurse, technician, or nursing assistant and community health agents; and there may be a linked oral health team, formed by a dentist, dental assistant, and dental hygiene technician. The primary health care coverage is estimated by the percentage of the population covered by teams of the Family Health Strategy and by equivalent traditional Primary Care teams in relation to the estimated population [32].

Table S3: Number of human cases of rabies and dog cases, Brazil, 1973 to 2022

Year	Total number of human cases	Human cases by dogs	Human cases by cats	Human cases by bats	Human cases by NHPs and wild canids	Human cases unknown species	Total number of dog cases
1973	107	0	0	0	0	107	1438
1974	129	0	0	0	0	129	12475 *
1975	120	0	0	0	0	120	13710 *
1976	99	0	0	0	0	99	4735
1977	141	0	0	0	0	141	5231
1978	141	0	0	0	0	141	3478
1979	190	0	0	0	0	190	5231
1980	168	147	5	1	3	12	4500
1981	139	119	3	1	2	14	2955
1982	125	110	1	5	0	9	2275
1983	101	88	4	4	1	4	1216
1984	87	75	4	2	1	5	874
1985	52	37	0	8	1	6	496
1986	38	28	1	4	3	2	462
1987	54	42	2	3	2	5	441
1988	37	30	1	4	1	1	464
1989	58	44	1	2	4	7	594
1990	73	50	2	11	2	8	823
1991	70	49	3	8	5	5	1068
1992	60	38	2	13	2	5	850
1993	50	38	4	5	2	1	674
1994	22	16	1	3	0	2	744
1995	31	26	1	2	0	2	1014
1996	25	20	1	1	1	0	1058
1997	25	18	3	1	1	0	1454
1998	29	20	2	4	3	0	1737
1999	25	23	0	2	0	0	1231

2000	26	24	1	0	1	0	921
2001	21	18	1	0	2	0	895
2002	10	6	0	3	0	1	635
2003	17	14	0	3	0	0	271
2004	28	5	1	22	0	1	169
2005	39	1	0	42	1	0	91
2006	10	6	0	2	0	1	81
2007	1	1	0	0	0	0	83
2008	3	0	0	2	1	0	34
2009	2	2	0	0	0	0	26
2010	3	1	0	0	1	1	18
2011	2	2	0	0	0	0	73
2012	5	2	0	1	2	0	83
2013	5	3	0	0	2	0	32
2014	0	0	0	0	0	0	16
2015	2	1	1	0	0	0	83
2016	2	0	1	1	0	0	11
2017	6	0	1	5	0	0	14
2018	11	0	0	11	0	0	14
2019	1	0	1	0	0	0	16
2020	2	0	0	1	1	0	12
2021	1	0	0	0	1	0	12
2022	5	0	0	4	0	1	7

Sources: All human cases by the transmitting animal from 1986 to 2022 are available on tables and figures in the Ministry of Health webpage [34]. From 1973 to 1985, the source was Schneider (1990) based on the Ministry of Health [35]. All canine cases of rabies from 2002 to 2020 are publicly available on the government website [34]; before that, the source was Schneider (1990) and internal reports from the Ministry of Health [35].

* The number of dog cases in these two years could not be confirmed; this could be a reflection of adjusting the rabies surveillance system being developed in Brazil.

Figure S1. Conceptual model of human rabies transmission by hematophagous bats (A) and epidemiological components of the mathematical model for human cases of rabies virus transmission (B)

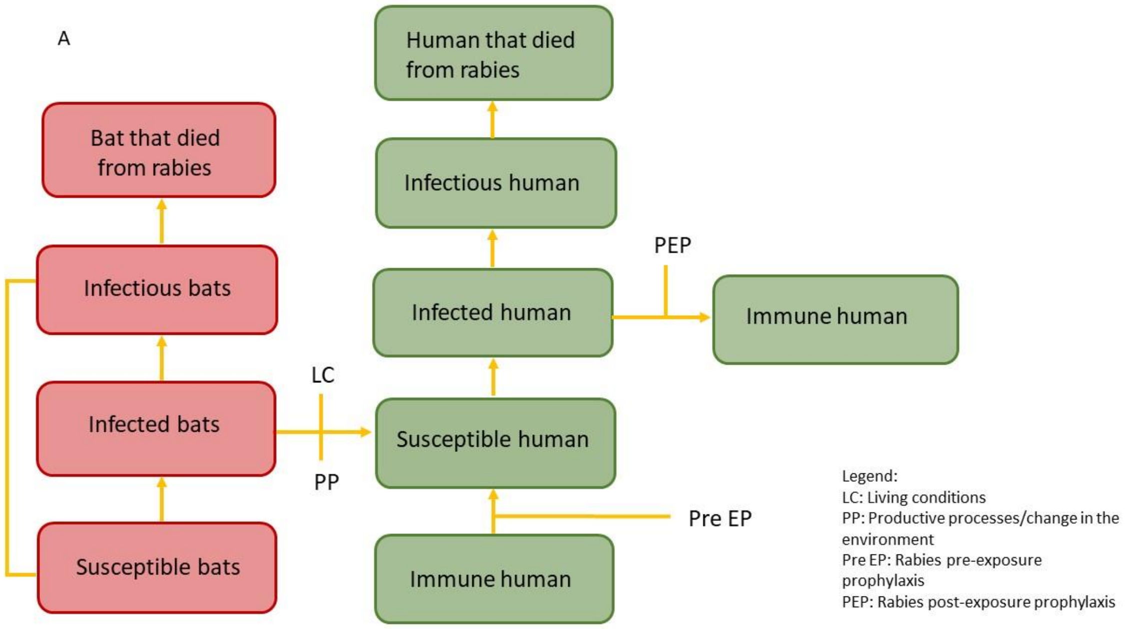

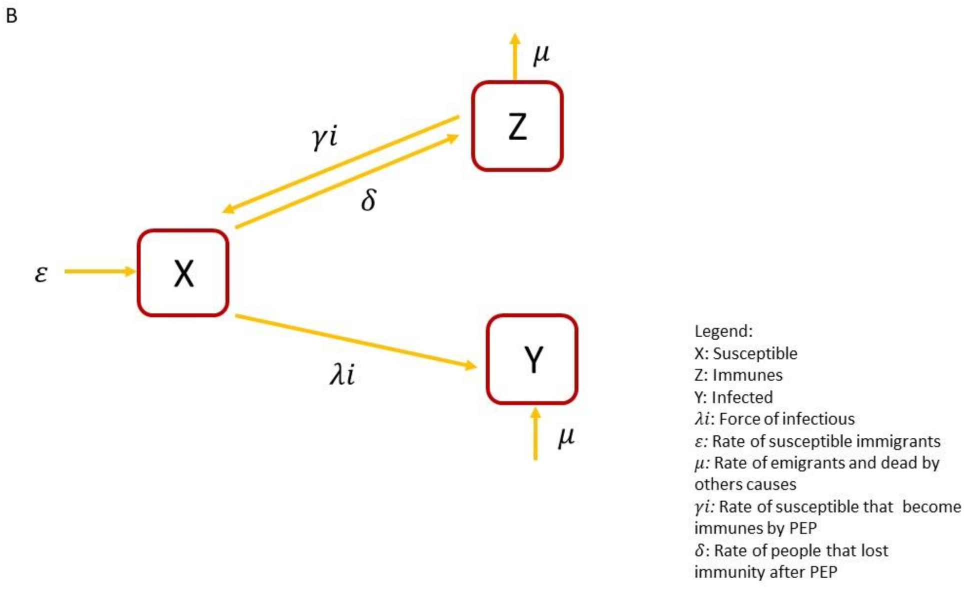

Table S4: Number of rabies cases in dogs reported and variants detected, by region and state, Brazil, 2010-2022

**Region**	**2010**	**2011**	**2012**	**2013**	**2014**	**2015**	**2016**	**2017**	**2018**	**2019**	**2020**	**2021**	**2022**	**Total (%)**
State														
**North**	**1**	**7**					**1**		**1**	**1**				**11**
Acre	-													
Amapá	-						1 [b]							1
Amazonas	-													
Pará	1 [a]	7							1 [b]					9
Rondônia	-									1				1
Roraima	-													
Tocantins	-													
**Northeast**	**17**	**65**	**79**	**32**	**13**	**11**	**7**	**12**	**10**	**11**	**10**	**10**	**3**	**280**
Alagoas	-										1 [c]			1
Bahia	-	1	1					3 [c]		3 [c]	3 [c]	1 [b]		12
Ceará	4	3	6	2 [a c]		3 [a c]		3 [b c]	3 [c]	2 [c]	2 [c]	1	1	30
Maranhão	2	55	69	29	11 [a c]	2 [a]	1	4 [a c]	4 [c]	2				179
Paraíba	2							1 [c]			1 [c]	1 [c]		5
Pernambuco	4	2	2				4 [c]		2 [c]	2 [c]	2 [c]	4 [c]	1	23
Piauí	2	3									1 [b]	2 [c]		8
Rio Grande do Norte	3	1	1	1	1	6 [c]	1 [c]		1 [c]	2 [c]		1 [c]	1 [c]	19
Sergipe	-				1 [c]		1 [b]	1 [b]						3
**Southeast**	**-**		**3**		**2**	**1**	**1**	**1**	**2**	**2**		**2**	**3**	**17**
Espírito Santo	-													
Minas Gerais	-		1 [a]									1 [b]	1	3
Rio de Janeiro	-											1 [b]	1	2
São Paulo	-		2 [b]		2 [b]	1 [b]	1 [b]	1 [b]	2 [b]	2 [b]			1 [b]	12
**South**	**-**						**1**				**2**			**3**
Paraná	-										2 [b]			2
Rio Grande do Sul	-													
Santa Catarina	-						1 [b]							1
**Central-West**	**-**													**80**
Federal District	-													
Goiás	-				1 [b]									1
Mato Grosso	-								1 [b]	2 [b]			1	4

Mato Grosso do Sul	-	1 [b]	1 [d]			71 [d]	1 [d]	1 [b]						75
**Total**	**18**	**73**	**83**	**32**	**16**	**83**	**11**	**14**	**14**	**16**	**12**	**12**	**7**	**391**

Legend: [a] Variant AgV2 (domestic dog), [b] Variant AgV3 (hematophagous bat), [c] Variant AgV2* (*Cerdocyon thous*), [d] Variant AgV1 (domestic dog)

Sources: Health Ministry of Brazil [34]

Table S5: Descriptive analysis of the explanatory variables

Variable	Minimum	Median	Maximum
Temperature	19.48	24.91	27.81
Precipitation	371	903	2645
Tree loss %		94.16	100
GDP per capita (Reais)	4625	8901	220213
Gini index	0.3684	0.5305	0.7972
Rural population%	0	47	92
Population density	0.87	29.41	8531.64
Primary healthcare coverage	45.5	100	100
Dog vaccination coverage	18.48	90.93	100

Table S6: Statistical analysis (a. univariate, b. multivariate final model) of possible drivers for cases of human rabies in the Northeast Region, 2010-2022

a. Univariate		
Variable	Category	OR (95%CI
Major habitat type (biome)	Tropical vs Non-tropical	1.092 (0.389 – 2.945)
Temperature	Continuous	1.703 (1.199 – 2.598)
Precipitation	Continuous	1.001 (1.000 – 1.002)
Treeloss %	Continuous	1.001 (0.982 – 1.026)
GDP per cap	Continuous	1.000 (0.999 – 1.000)
Gini Index	>0.5 (vs below)	2.444 (0.679 – 15.6005)
Population density 2020	Continuous	1.000 (0.999 – 1.001)
Rural population %	Continuous	0.977 (0.952 - 1.002)
Primary healthcare coverage	Continuous	0.957 (0.928 – 0.998)
Dog vaccination coverage	Continuous<br>Equal or >80% (vs below)	1.063 (1.000 – 1.149)<br>3.378 (0.681 – 61.194)
b. Multivariate final model		
Temperature	Continuous	1,739 (1.181 – 2.744)
Primary healthcare coverage	Continuous	0.947 (0.915 – 0.987)
